# Adipokines and Bacterial Metabolites: A Pivotal Molecular Bridge Linking Obesity and Gut Microbiota Dysbiosis to Target

**DOI:** 10.3390/biom13121692

**Published:** 2023-11-23

**Authors:** Teva Turpin, Katy Thouvenot, Marie-Paule Gonthier

**Affiliations:** Université de La Réunion, INSERM, UMR 1188 Diabète Athérothrombose Thérapies Réunion Océan Indien (DéTROI), 97410 Saint-Pierre, La Réunion, France; teva.turpin@univ-reunion.fr (T.T.); katy.thouvenot@univ-reunion.fr (K.T.)

**Keywords:** adipokines, bacterial metabolites, gut microbiota dysbiosis, insulin resistance, obesity, vascular disorder

## Abstract

Adipokines are essential mediators produced by adipose tissue and exert multiple biological functions. In particular, adiponectin, leptin, resistin, IL-6, MCP-1 and PAI-1 play specific roles in the crosstalk between adipose tissue and other organs involved in metabolic, immune and vascular health. During obesity, adipokine imbalance occurs and leads to a low-grade pro-inflammatory status, promoting insulin resistance-related diabetes and its vascular complications. A causal link between obesity and gut microbiota dysbiosis has been demonstrated. The deregulation of gut bacteria communities characterizing this dysbiosis influences the synthesis of bacterial substances including lipopolysaccharides and specific metabolites, generated via the degradation of dietary components, such as short-chain fatty acids, trimethylamine metabolized into trimethylamine-oxide in the liver and indole derivatives. Emerging evidence suggests that these bacterial metabolites modulate signaling pathways involved in adipokine production and action. This review summarizes the current knowledge about the molecular links between gut bacteria-derived metabolites and adipokine imbalance in obesity, and emphasizes their roles in key pathological mechanisms related to oxidative stress, inflammation, insulin resistance and vascular disorder. Given this interaction between adipokines and bacterial metabolites, the review highlights their relevance (i) as complementary clinical biomarkers to better explore the metabolic, inflammatory and vascular complications during obesity and gut microbiota dysbiosis, and (ii) as targets for new antioxidant, anti-inflammatory and prebiotic triple action strategies.

## 1. Introduction

The World Health Organization (WHO) has recognized obesity as a chronic metabolic disease and has defined it as an abnormal and excessive fat accumulation that can be detrimental to health. Obesity is considered as one of the most careless public health problems and has reached epidemic proportions in low- and middle-income countries, especially in urban areas [1]. The fundamental cause of obesity is an energy imbalance between energy expenditure and intake, despite other factors that may be involved such as genetic status or drug side-effects [2]. Consequently, obesity constitutes an important risk factor for metabolic and chronic disorders such as dyslipidemia, insulin resistance and related type 2 diabetes (T2D), hypertension, cardio- and neuro-vascular diseases, and even several types of cancers [3]. The target organ characterizing the adiposity degree of individuals is the adipose tissue. During obesity, this organ plays a crucial role at cellular and molecular levels in insulin resistance, T2D onset and their vascular complications.

Adipose tissue is a connective tissue rich in extracellular matrix and composed of specific cells called adipocytes, originating from the differentiation of preadipocyte precursors. It also contains the stromal vascular fraction comprising fibroblasts, macrophages and other immune cells, endothelial cells and nerve tissue [4]. The primary function of adipocytes is to store energy as triglycerides that are synthetized through the metabolism of fatty acids and glycerol, itself generated from glucose. Physiologically, the proportion of the adipose tissue can account for 10 to 30% of a subject’s total body weight [5]. The discovery of adipocyte-secreted leptin and adiponectin, three decades ago, demonstrated that adipose tissue also acts as an endocrine organ [6,7]. Indeed, adipose tissue secretes several molecules called adipokines, such as leptin, adiponectin, resistin, cytokines and chemokines including interleukin (IL)-6 and monocyte chemoattractant protein-1 (MCP-1), respectively, and other factors like plasminogen activator inhibitor 1 (PAI-1) [8]. Due to its metabolic, immune and endocrine functions, adipose tissue is strongly involved in the pathology of obesity.

During obesity, the positive energy balance mainly caused by overfeeding and sedentary lifestyle leads to adipose tissue expansion. This adipose tissue enlargement induced by hypertrophy (increase in adipocyte size) and hyperplasia (increase in preadipocyte number) of adipose cells is associated with morphological and functional changes, contributing to adipose tissue dysfunction [9]. In particular, the dysregulation of metabolic and endocrine functions of adipose cells generates (1) an imbalance between the overproduction of reactive oxygen species (ROS) and the endogenous defense system composed of major antioxidant enzymes, leading to oxidative stress [10]; and (2) an imbalance between the up-regulated production of pro-inflammatory adipokines and the down-regulated synthesis of anti-inflammatory adipokines, causing a pro-inflammatory status [11]. Both oxidative stress and inflammation contribute to insulin resistance of the adipose tissue and other organs like the skeletal muscles, thereby promoting a diabetic status in patients with obesity [12].

During the last two decades, new evidence has emerged concerning a link between gut microbiota dysbiosis and adipose tissue dysfunction in obesity. Data from the literature reported that gut bacteria composition is changed in patients with obesity [13] as well as in mouse models of high-fat diet (HFD)-induced obesity [14]. In particular, the intestinal bacteria dysbiosis occurring during obesity increases the gut barrier “leaking”, contributing to blood translocation of bacterial components such as lipopolysaccharides (LPSs) derived from Gram-negative bacteria like *Escherichia coli* (*E. coli*). Concordantly, *E. coli* LPS levels are elevated in plasma during obesity, characterizing an endotoxemia, and may exacerbate inflammation and insulin resistance in adipose tissue [15]. Other bacterial metabolites produced from the catabolism of dietary components by the colonic bacteria have also been associated with metabolic and vascular health. These metabolites include short-chain fatty acids (SCFAs), trimethylamine (TMA) metabolized into trimethylamine-oxide (TMAO) in the liver and indole derivatives. Nevertheless, there is a lack of data regarding the modulation of the production of these gut bacteria-derived metabolites and their impact on adipose tissue function and adipokine production in obesity.

This review aims to provide an overview of the links between adipokines and gut bacteria-derived metabolites in the context of obesity and gut microbiota dysbiosis. Firstly, six key adipokines known to be deregulated in obesity and related metabolic and vascular complications are described, namely adiponectin, leptin, resistin, IL-6, MCP-1 and PAI-1. Secondly, the modulation of these adipokines by gut bacteria-derived metabolites and their suggestive molecular links in mechanisms underlying oxidative stress, inflammation and vascular dysfunction occurring in obesity are presented, providing evidence for the relevance of adipokines and bacterial metabolites as useful complementary clinical biomarkers and as targets for nutritional/pharmacological strategies to develop.

## 2. Adipokine Production by Adipose Tissue and Relationship with Obesity

Adipose tissue is a very rich tissue in terms of cellular diversity, and adipocytes constitute the specific functional adipose cells. It is commonly accepted that there are three types of adipocytes, namely the white adipocytes, the beige/brite adipocytes and the brown adipocytes, with different anatomical locations [16,17]. This review is preferentially focusing on the white adipose tissue (WAT). WAT is implicated in energy storage via the accumulation of triglycerides in lipid droplets of adipocytes, thermal insulation and mechanical protection that is important for resisting infection and injury [18]. Adipose tissue energy storage is dependent on insulin which is responsible for 5% of insulin-mediated glucose uptake in normo-weight subjects and 20% in patients with obesity [19]. However, if WAT was long-known as a storage organ, the discovery of adipocyte-derived hormones such as leptin and adiponectin revealed that WAT also acts an endocrine organ [6,7]. Indeed, adipocytes, preadipocytes and some cells of the stromal vascular fraction produce several adipokines that act locally (autocrine/paracrine) and systemically (endocrine). Adipose tissue dysfunction and adipokine dysregulation are thought to be responsible for and/or contribute to the increased risk of obesity, and related metabolic and vascular complications. Of note, during obesity, up-regulated levels of pro-inflammatory adipokines like IL-1β, IL-6, MCP-1, tumor necrosis factor-α (TNF-α), PAI-1, leptin and resistin, and down-regulated levels of anti-inflammatory adipokines such as adiponectin and IL-10, lead to a chronic state of low-grade inflammation. This pro-inflammatory status promotes the development of insulin resistance and T2D, atherosclerosis and other vascular disorders [20,21]. The following part of the review describes the key roles of adiponectin, leptin, resistin, IL-6, MCP-1 and PAI-1 recognized to be involved in the physiopathology of obesity and cited as relevant targets for limiting metabolic and vascular complications in obesity.

### 2.1. Adiponectin

**Figure 1:**
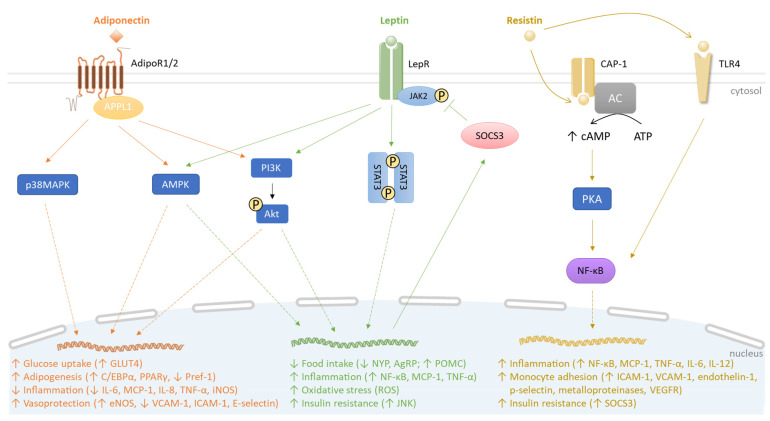
Comparative signaling pathways mediated by adiponectin, leptin and resistin. Adiponectin, leptin, and resistin bind to their receptors AdipoR1/2, LepR, and CAP-1, respectively. Resistin also binds to TLR4. AdipoR1/2 recruits APPL1 and activates p38MAPK, AMPK and PI3K/Akt pathways, increasing (↑) glucose uptake and adipogenesis. Simultaneously, adiponectin decreases (↓) pro-inflammatory response and promotes vasoprotection (orange). The engagement of leptin with LepR leads to JAK2 activation, initiating STAT3 dimerization. JAK2/STAT3 induction correlates with SOCS3 production, which, in turn, mediates a negative feedback on JAK2/STAT3 signaling. Similar to AdipoR1/2, LepR can induce AMPK and PI3K/Akt pathways. In contrast to adiponectin, leptin causes a pro-inflammatory response along with oxidative stress. Moreover, leptin contributes to insulin resistance by stimulating JNK activity (green). Under resistin action, CAP-1 induces the production of cAMP by the adenylyl cyclase (AC) that activates PKA. Interconnected PKA/TLR4 signaling pathways converge to recruit NF-κB, leading to inflammation and insulin resistance (yellow).

Adiponectin is an adipokine of approximately 30 kDa, produced by the white adipocytes. It circulates in plasma in three major oligomeric forms, namely a low-molecular-weight, a middle-molecular-weight, or a high-molecular-weight (HMW) adiponectin. The HMW form is the most biologically active [22]. Adiponectin has pleiotropic beneficial effects on both adipose tissue and vascular endothelial cells through its anti-inflammatory, antioxidant, anti-apoptotic, vasodilator and anti-thrombotic activities [23]. Adiponectin also improves insulin sensitivity and acts locally on adipose tissue in an autocrine manner via its receptors to reduce the release of pro-inflammatory cytokines from adipocytes and surrounding stromal vascular cells (e.g., IL-6 and MCP-1) [24]. Adiponectin signaling relies on two atypical seven-transmembrane receptors, adiponectin receptor 1 (AdipoR1) and adiponectin receptor 2 (AdipoR2), to initiate the activation of several intracellular signaling cascades such as AMP-activated kinase (AMPK) and p38 mitogen activated protein kinase (MAPK) pathways, leading to its diverse biological activities on the metabolic, inflammatory and vascular status (Figure 1). Adiponectin gene expression is regulated by three key adipogenic transcription factors, namely CCAAT/enhancer-binding protein (C/EBP) α, peroxisome-proliferator activated receptor (PPAR) γ and sterol-regulatory-element-binding protein (SREBP)1c [25]. It is established that proinflammatory cytokines, including TNF-α and IL-6, suppress adiponectin gene expression and this effect may be mediated through c-Jun N-terminal kinase (JNK) and p44/42 MAPK (Erk 1/2) [26,27]. Oppositely, adiponectin gene expression is up-regulated by insulin sensitizers and anti-diabetic drugs including thiazolidinediones, metformin and sulfonylurea, as well as some micronutrients like polyphenols provided by fruits and vegetables [28,29].

In humans, adiponectin accounts for as much as 0.01% of total plasma proteins. Average levels of plasma adiponectin range from 3 to 30 μg/mL [30], and low plasma adiponectin levels (<4 μg/mL) are closely associated with obesity-linked metabolic and cardiovascular disorders [31,32]. Adiponectinemia inversely reflects the total body fat mass, given that the higher the concentration of plasma adiponectin, the lower the percentage of body fat. Moreover, adiponectinemia is directly associated with insulin sensitivity and can be considered as a biomarker of adipose tissue health [22,32,33]. Interestingly, weight-loss mediated by bariatric surgery allows a sustained increase in adiponectinemia [34]. In parallel, epidemiological studies suggest that hypoadiponectinemia is associated with endothelial dysfunction, hypertension, increased risk of atherosclerosis and coronary artery disease [35,36].

To elucidate the mechanisms of action of adiponectin, several animal and cellular models were developed. In particular, in apolipoprotein E-deficient (ApoE^−/−^) mice exhibiting deregulated cholesterol metabolism and atherosclerotic plaques, adiponectin administration attenuated metabolic and vascular disorders, by increasing the production of the anti-inflammatory cytokine IL-10 and the endothelial nitric oxide synthase (eNOS) responsible for the synthesis of the vasodilator NO. Oppositely, adiponectin reduced the production of pro-inflammatory mediators like TNF-α, IL-6 and vascular cell adhesion molecule-1 (VCAM-1), leading to decreased endothelial inflammation. Additionally, adiponectin injection counteracted the nuclear factor-κappa B (NF-κB) signaling pathway activation [37]. Meanwhile, in ApoE^−/−^ mice, adiponectin exerted an antioxidant action by inhibiting inducible NO synthase (iNOS) involved in inflammation and oxidative stress [38]. In in vitro studies, 3T3-L1 preadipocyte transduced cell lines, expressing adiponectin, exhibited more prolonged and robust expression of genes coding for the pro-adipogenic transcription factors C/EBPα, PPARγ and SREBP1c. Furthermore, adiponectin increased insulin sensitivity and glucose uptake by enhancing glucose transporter 4 (GLUT4) gene expression and recruitment to the plasma membrane of adipocytes [39]. Otherwise, in an adenovirus-infected 3T3-L1 preadipocyte model producing adiponectin and co-cultured with mature adipocytes exposed to LPS, adiponectin overexpression lowered MCP-1, IL-6, IL-8 and TNF-α production, and increased preadipocyte differentiation with an up-regulation of C/EBPα and PPARγ pathways, while down-regulating preadipocyte factor-1 (Pref-1) signaling that acts as an adipogenesis inhibitor [40]. In in vitro models of endothelial cells exposed to TNF-α, adiponectin exposure promoted NO production by inducing eNOS phosphorylation via the AMPK pathway. It also dose-dependently inhibited TNF-α–induced monocyte adhesion by reducing the production of VCAM-1, intercellular adhesion molecule (ICAM)-1 and E-selectin, involved in leukocyte diapedesis [41,42]. Altogether, these preclinical and in vitro findings demonstrate that, mechanistically, adiponectin may act through anti-inflammatory, antioxidant, insulin-sensitizer, pro-adipogenic and endothelium-protective effects under inflammatory conditions, helping to limit metabolic and vascular complications during obesity (Figure 1).

### 2.2. Leptin

Leptin is an adipokine of 16 kDa produced primarily by WAT. It is essential for body weight control by regulating food intake and energy expenditure via the activation of leptin receptors (LepR) in the hypothalamus [43]. Six LepRs have been identified, with the long isoform LepRb being highly expressed in the hypothalamus. Leptinemia is up-regulated upon food intake and in patients with obesity, and decreased during fasting or weight loss. Additionally, inflammatory stimuli such as LPS and TNF-α increase leptin production in adipose tissue, and, thus, the plasma leptin level [44,45]. After binding to LepRb, leptin activates the pathway depending on the Janus kinase 2 (JAK2) and the signal transducer and activator of transcription 3 (STAT3), and induces the expression of the suppressor of cytokine signaling 3 (SOCS3), a negative regulator of leptin signaling (Figure 1). The leptin-mediated pathway leads to the enhanced production of the hypothalamic anorexigenic neuropeptide proopiomelanocortin (POMC) while down-regulating the synthesis of the orexigenic neuropeptide Y (NYP) and agouti protein (AgRP) [46]. Thus, leptin behaves as a satiety hormone, acting through feedback to the hypothalamus to regulate appetite, according to the body mass and the nutritional status.

During obesity, leptin resistance occurs due to leptin’s inability to reach the target cells, reduced LepR gene expression or disturbed LepR signaling [47]. Patients with obesity exhibit higher plasma levels of leptin (average rate of 36 ng/mL) than those measured in normo-weight subjects (8 ng/mL). A positive correlation between leptinemia and total body fat mass has been clearly reported [48]. In obese individuals, hyperleptinemia may act as an immunomodulatory and pro-inflammatory signal. Indeed, high levels of leptin enhance the production of pro-inflammatory cytokines, and in a reciprocal manner, pro-inflammatory mediators such as IL-6, TNF-α, IL-1β or LPS up-regulate leptin secretion by adipocytes, and consequently leptinemia [49,50]. The adiponectin/leptin ratio was suggested as an adipose tissue dysfunction marker and correlates with insulin resistance more closely than adiponectin or leptin alone, or even the HOMA-IR (homeostatic model assessment for insulin resistance) index which evaluates insulin resistance [51]. Of note, bariatric surgery improves metabolic profile and decreases leptinemia in patients with obesity [52]. Leptin’s role in the cardiovascular system remains controversial. Nevertheless, given the contribution of hyperleptinemia to the low-grade systemic inflammation during obesity, it is suggested that high leptin levels may predispose obese patients to a cardiovascular disease risk [53].

To understand leptin’s mechanisms of action, preclinical and cellular studies were conducted. In animal models, HFD-induced obesity reduced leptin sensitivity and increased food intake [54]. In mouse models of partial leptin deficiency, it was shown that a reduced leptin level counteracted diet-induced obesity, adipose tissue inflammation and enhanced insulin sensitivity and glucose tolerance [55]. In parallel, in in vitro cellular studies, leptin treatment of macrophages promoted a pro-inflammatory response with elevated secreted levels of cytokines, via induction of p38 MAPK and JNK signaling pathways [56]. Moreover, leptin was found to act as a potent chemoattractant for monocytes and macrophages, via LepRb-mediated activation of MAPK, JAK/STAT and phosphatidylinositol 3-kinase (PI3K) pathways [57]. Chronic leptin treatment also induced preadipocyte differentiation and an adipocyte pro-inflammatory response marked by increased TNF-α release [58]. Concomitantly, in a human umbilical vein endothelial cell model, leptin exposure increased the production of ROS, which activated the JNK pathway and increased DNA-binding activity of the pro-inflammatory transcription factors NF-κB and activator protein 1 (AP-1), leading to MCP-1 overproduction. Taken together, these data demonstrate that leptin, in contrast to adiponectin, promotes adipose tissue and vascular inflammation and oxidative stress, which may contribute to the development of insulin resistance and vascular disorders during obesity (Figure 1).

### 2.3. Resistin

Resistin is an approximately 12 kDa polypeptide that is secreted by preadipocytes, adipocytes and macrophages in humans, and is strongly related to obesity and insulin resistance [21,59]. Pro-inflammatory stimuli like LPS, TNF-α, IL-6 and IL-1β have been shown to positively regulate the gene expression and secretion of resistin through the NF-ĸB pathway [60,61]. Oppositely, the PPARγ pathway, exerting anti-inflammatory and insulin-sensitizing action, down-regulates resistin production [62]. As illustrated in Figure 1, resistin mediates its biological effects via the direct activation of the adenylyl cyclase-associated protein 1 (CAP-1). CAP-1 consists of three domains comprising an N-terminal domain associated with adenylate cyclase (AC), a central Src homology 3 (SH3) domain and an actin binding C-terminal domain. Binding of resistin to CAP-1 is related to the SH3 domain which initiates signaling through AC. This event leads to the increased level of cAMP that activates the protein kinase A (PKA) and subsequently NF-κB, resulting in pro-inflammatory cytokine production [63]. Toll-like receptor 4 (TLR4), belonging to the innate immunity receptors family, has also been reported to bind human resistin. The resistin-mediated TLR4 pathway could mediate NF-κB activation and some of the pro-inflammatory effects of resistin, in particular via the secretion of MCP-1, TNF-α, IL-6 and IL-12 in monocytes and macrophages [64] (Figure 1).

Obese patients who have greater infiltration of macrophages in adipose tissue exhibit higher levels of resistin in adipose tissue samples as well as higher resistinemia (average rate of 15 ng/mL) than that detected in lean individuals (<10 ng/mL) [59,65]. High plasma resistin levels were also found to positively correlate with an increased risk of all-cause and cardiovascular death [66]. Interestingly, bariatric surgery decreases plasma resistin concentration in obese patients [67].

Animal and cellular studies helped to explore the mechanisms underlying resistin’s biological effects. Of note, it was reported that HFD-induced obese mice exhibited increased plasma levels of resistin while immunoneutralization of circulating resistin in these animals improved insulin resistance [68]. Concordantly, elevated resistinemia was depicted in *ob*/*ob* obese mice [69]. In a transgenic mouse model expressing human resistin exclusively, the adipose tissue inflammation was exacerbated, and lipolysis and free fatty acid accumulation increased, contributing to insulin resistance [70]. It was also demonstrated that resistin interfered with the insulin signaling cascade by deregulating the activation of insulin receptor substrate (IRS)-1, PI3K and protein kinase B/Akt, and by inducing the production of the suppressor of cytokine signaling 3 (SOCS3) known to inhibit insulin signaling in the liver, skeletal muscles and WAT [71,72]. In vitro studies using 3T3-L1 adipocyte cell line showed that resistin neutralization by resistin antiserum enhanced insulin-stimulated glucose uptake and decreased insulin resistance [68]. In endothelial cells cultured in the presence of resistin, the production of endothelin-1 (ET-1) vasoconstrictor was elevated. Moreover, resistin increased the production of several pro-inflammatory mediators such as MCP-1 and adhesion molecules including ICAM-1 and VCAM-1, leading to monocyte adhesion [73,74] (Figure 1). These findings show that, conversely to adiponectin but similarly to leptin, resistin acts as a mediator of inflammation, insulin resistance and vascular dysfunction during obesity.

### 2.4. IL-6

**Figure 2:**
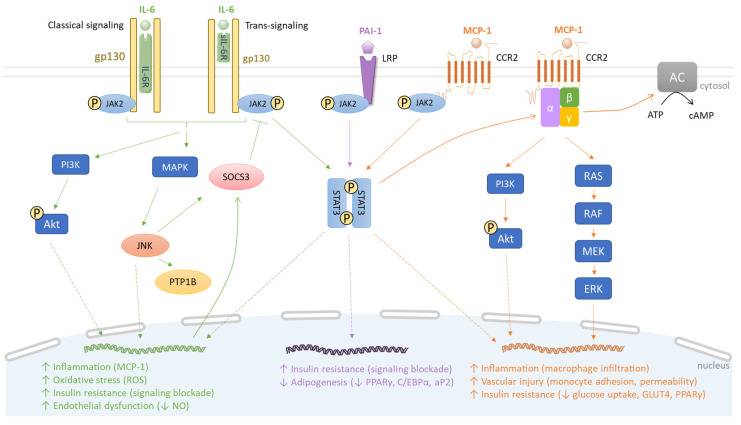
Comparative signaling pathways of IL-6, PAI-1 and MCP-1. IL-6, PAI-1 and MCP-1 bind to IL-6R/sIL-6R, LRP and CCR2, respectively. Subsequently, JAK2 phosphorylates two STAT3 molecules, resulting in STAT3 dimerization, DNA binding and transcription of target genes. For instance, in the IL-6 signaling pathway, this leads to SOCS3 induction which enacts a negative feedback on JAK2/STAT3 signaling. The interaction of IL-6 with IL-6R/sIL-6R activates PI3K/Akt and MAPK pathways that induce JNK, PTP1B and SOCS3, thus promoting inflammation, oxidative stress, insulin resistance and vascular disorder (green). The PAI-1 signaling pathway contributes to insulin resistance and suppresses adipogenesis (purple). Via the MCP-1 signaling pathway, the dimerized STAT3 activates G proteins, subsequently triggering PI3K/Akt, RAS/RAF/MEK/ERK and adenylyl cyclase (AC) pathways. This causes macrophage infiltration and insulin resistance (orange).

IL-6 is a cytokine of 21–28 kDa produced by different tissues. WAT constitutes a major source of circulating IL-6, up to 35% of basal circulation in humans [75]. IL-6 gene expression is mainly regulated by NF-ĸB and JNK/AP-1 pathways. These signaling pathways are induced by LPS, free fatty acids, high glucose conditions, pro-inflammatory cytokines (e.g., IL-6, TNF-α) and ROS [76,77]. Recently, it was demonstrated that interaction between IL-1β and TNFα promotes the elevation of IL-6 gene expression via the involvement of C-AMP Response Element-binding protein (CREB) acting as a transcription factor, and histone 3 lysine 14 (H3K14) acetylation on the CRE locus in adipocytes from obese patient tissue but not lean subject tissue [78]. IL-6 signaling can be mediated through a canonical receptor (classical signaling) or noncanonical receptor (trans-signaling) (Figure 2).

The classical signaling requires IL-6 binding to the subunit α of the IL-6 receptor (IL-6R) on the plasma membrane. This interaction induces a dimerization with IL-6R subunit β (gp130), initiating intracellular signaling through JAK/STAT, MAPK and PI3K/Akt pathways, depending on the cellular types [79]. In the trans-signaling mode, IL-6 binds to a soluble IL-6R (sIL-6R), originating from a spliced IL-6R mRNA and complexing with gp130 [80]. Then, both classical signaling and trans-signaling mediate common intracellular events. In insulin-dependent tissues like WAT, the phosphorylation of STAT3 and dimerization of phosphorylated STAT3 promote STAT3 translocation into the nucleus to induce the production of SOCS3. In parallel, the MAPK pathway activates JNK, which also enhances SOCS3 production and protein-tyrosine phosphatase 1B (PTP1B) activity, causing the phosphorylation of a serine residue of IRS-1 and insulin receptor-mediated pathway blockade. In human vascular endothelial cells, IL-6 trans-signaling induces the release of MCP-1 dependent on the simultaneous activation of the JAK/STAT3 and PI3K/Akt pathways (Figure 2).

During obesity, IL-6 production is enhanced. Plasma levels increase from an average rate of 2.5 pg/mL in lean subjects to 4.4 pg/mL in patients with obesity [81]. Bariatric surgery-mediated fat mass loss significantly decreases the circulating concentration of IL-6 [82]. In target tissues, IL-6 induces the production of various acute inflammation-phase proteins including C-reactive protein (CRP), which is generated by the liver and used as a clinical marker of systemic inflammation [83]. IL-6 also causes major deregulations in glucose and lipid metabolism with the decrease in hepatic insulin sensitivity, insulin-dependent hepatic glycogen synthesis or adipocyte glucose uptake and triglyceride storage [84,85]. In endothelial cells, IL-6 is implicated in ROS synthesis and NO production reduction [86], suggesting its contribution to the development of insulin resistance and atherosclerosis.

In animal models of obesity used to explore IL-6 biological functions, a paradoxical role of IL-6 signaling in modulating inflammation and metabolism is reported. Indeed, the trans-signaling is considered to induce a pro-inflammatory response. Increased WAT inflammation in HFD-fed mice may be due to impaired anti-inflammatory effects of canonical IL-6 signaling and/or a shift to pro-inflammatory trans-signaling, resulting in macrophage accumulation in WAT. Blocking the IL-6 trans-signaling prevents macrophage infiltration in WAT during HFD-induced obesity, but does not improve insulin resistance [87]. In parallel, blocking IL-6 trans-signaling in the paraventricular nucleus of the hypothalamus enhances feeding and glucose intolerance [88]. Han et al. [89] showed that adipocyte-derived IL-6 increases adipose tissue macrophage infiltration, whereas myeloid cell- and muscle-derived IL-6 inhibits it, suggesting that IL-6 signaling is regulated in a depot and cell-specific manner. Recently, it has been reported that IL-6 deletion in adipocytes from HFD-induced obesity and *ob*/*ob* mouse models had no effect on glucose tolerance or fasting hyperinsulinemia [90]. Of note, IL-6-deficient mice developed late-onset obesity and systemic glucose intolerance [91]. Accumulating evidence suggests that IL-6 may contribute to the maintenance of normal systemic glucose metabolism and that the increased circulating IL-6 concentrations during obesity may actually be an adaptive mechanism to resist obesity-associated glucose intolerance [92,93].

### 2.5. MCP-1

MCP-1, also called the chemokine (C-C motif) ligand 2 (CCL2), is a protein of 9–15 kDa secreted by endothelial, epithelial, smooth muscle, mesangial, astrocytic and immune cells, as well as preadipocytes and adipocytes. Monocytes and macrophages are major sources of MCP-1 [94]. MCP-1 is a chemoattractant protein that regulates migration and infiltration of monocytes/macrophages in response to a chemical stimulus (chemotaxis). Its gene expression is activated by various pro-inflammatory mediators such as IL-1β, TNF-α, IL-6 binding to the IL-6 soluble receptor, LPS, fatty acids and high glucose condition via MAPK, NF-κB and AP-1 pathways in several types of cells including adipocytes, aortic smooth muscle cells and cerebral endothelial cells [95,96,97,98]. In target cells, MCP-1 action is mediated by a specific chemokine receptor which belongs to the family of G-protein coupled receptors (Figure 2). Cells that express the distinct CC chemokine receptor 2 (CCR2) can migrate along the chemokine gradient upon MCP-1 activation. As a result of CCL2/CCR2 binding, JAK2 directly phosphorylates CCR2 at the Tyr^139^ position and promotes JAK2/STAT3 complex association to the receptor, activating several signaling cascades through G proteins and receptor coupling such as MAPK, PI3K and phospholipase C (PLC) pathways that lead to monocyte migration in adipose tissue as well as in the endothelium, contributing to inflammatory processes and vascular injury [99,100,101] (Figure 2).

During obesity, body fat mass is associated with higher adipose as well as circulating MCP-1 levels. Indeed, obese patients exhibit a higher MCP-1 plasma concentration (220 pg/mL) than that of lean subjects (100 pg/mL) [102]. Body weight reduction in people with severe obesity correlates with a decrease in circulating MCP-1 levels [103]. In obesity, MCP-1 is overproduced in the adipose tissue and its production allows the recruitment of macrophages that mediate inflammation, insulin resistance and T2D onset [104]. Clinical studies also support the causal link between the circulating MCP-1 levels during obesity and the risk of stroke, coronary artery disease and plaque vulnerability implicated in atherosclerosis, due to the enhanced monocyte adhesion to the endothelium and the combination of macrophages with accumulated oxidized low-density lipoproteins (LDL), leading to the formation of foam cells and atherosclerotic plaques [105,106,107,108].

In mechanistic studies using models of transgenic mice overexpressing MCP-1 in adipocytes and exposed to HFD-induced obesity, it was found that elevated production of MCP-1 by adipose cells contributed to macrophage infiltration and insulin resistance in adipose tissue. Moreover, the transgenic mice exhibited a reduced insulin-induced phosphorylation of Akt in both skeletal muscles and liver as well as hepatic steatosis [109,110]. In the 3T3-L1 adipocyte cell line exposed to MCP-1 for 72 h, there was a 30% decrease in insulin-stimulated glucose uptake. This correlated with reduced mRNA levels of the insulin-sensitive glucose transporter type 4 (GLUT4) and PPAR-γ, in favor of insulin resistance [111] (Figure 2). In an endothelial cell line model, MCP-1 treatment suppressed high-density lipoprotein (HDL) internalization and cholesterol efflux through CCR2 binding and activating the p42/44 MAPK signaling pathway. These data contributed to support the idea that MCP-1 may impair HDL function and promote atherosclerosis in coronary artery disease [112]. Thus, similarly to other adipokines like leptin, resistin and IL-6 overproduced by the adipose tissue during obesity, MCP-1 plays a causal role in inflammation, insulin resistance and atherosclerosis during obesity.

### 2.6. PAI-1

PAI-1 is a 52 kDa single-chain plasma glycoprotein produced by endothelial cells, preadipocytes, adipocytes and macrophages within the adipose tissue [113,114]. Its physiological role is to inhibit plasminogen activators (PA) such as tissue-type PA (tPA) and urokinase-type PA (uPA), preventing the conversion of plasminogen into active protease plasmin and leading to fibrinolysis inhibition. PAI-1 directly affects the formation and degradation of thrombus, meaning that PAI-1 acts as a procoagulant factor. PAI-1 production is regulated by the *SERPINE1* gene and can be induced in response to characteristic pro-inflammatory mediators including TNF-α, IL-6 and IL-1β [115]. Acute or chronic exposure of adipocytes to TNF-α leads to the activation of different signaling pathways. Indeed, acute exposure requires the activation of p44/42 MAPK and PKC for PAI-1 mRNA expression. In parallel, chronic exposure, besides the p44/42 MAPK and PKC pathways, leads to the activation of the p38 MAPK pathway, tyrosine kinases and a signaling cascade that involves PI3K and the transcription factor NF-κB. Chronic TNF-α exposure encountered in conditions such as obesity may activate alternative and/or compensatory signaling pathways for PAI-1 gene expression in adipocytes [115]. Similarly to TNF-α, IL-6 and IL-1β were shown to enhance the expression of the PAI-1 gene through the activation of MAPK and NF-ĸB pathways [116]. Conversely, the inhibition of TNFα signaling via adiponectin has been shown to reduce PAI-1 expression. It was also established that PAI-1 binding to lipoprotein receptor-related protein (LRP) 1 is able to trigger JAK/STAT1 signaling that promotes smooth muscle cells migration, intimal hyperplasia and vascular inflammation implicated in atherosclerosis and cardiovascular disease [117,118] (Figure 2). Interestingly, PAI-1 can form a complex with vitronectin which induces stabilization of the active conformation of PAI-1 in the circulation. PAI-1 suppresses insulin signaling by competing with integrin αvβ3 for vitronectin binding, known to potentiate the insulin receptor [119].

Clinical studies have demonstrated that the plasma PAI-1 level is higher in patients with obesity as compared to control individuals (55.1 vs. 10.4 ng/mL, respectively), and, inversely, is reduced with body weight loss. Concordantly, PAI-1 is positively associated with components of the metabolic syndrome (MS) [120]. Recently, a study compared plasma levels of cytokines every hour for 24 h between an insulin-sensitive group and an insulin-resistant group of people with obesity, and showed no difference in 24 h plasma concentration area under the curves for a battery of cytokines, except for PAI-1 [121]. Moreover, the plasma PAI-1 level was more elevated in the obese insulin-resistant group than that measured in the control group. Of note, patients with MS exhibit lower levels of adiponectin and higher PAI-1 levels as compared to lean subjects [122]. Interestingly, surgical fat removal or weight loss attributed to diet-mediated fat reduction is associated with a decreased plasma level of PAI-1 in obese patients [123].

In rodent models developed for exploring PAI-1’s physiological roles, specific PAI-1 overexpression in adipocytes causes insulin resistance, whereas specific PAI-1 deletion in adipocytes improves insulin action [124]. These results suggest that PAI-1 plays a causal role in the insulin resistance of adipocytes. In high-fat diet-induced obesity and insulin resistance mouse models, PAI-1 deficiency completely prevents the development of obesity and insulin resistance [125]. Furthermore, genetic or pharmacological reduction in PAI-1 activity led to a decrease in macrophage infiltration in WAT and an improvement of the metabolic status in a diet-induced obese mouse model [126,127]. In animal models used to investigate the vascular health, PAI-1 was associated with some controversial functions. Indeed, PAI-1 deficiency was shown to be protective [128] or play a detrimental role [129] in the development of atherosclerosis. Nonetheless, mouse models that overexpressed the PAI-1 gene exhibited elevated PAI-1 levels that were linked to thrombosis and cardiovascular disease development [130]. In a comparative study using cultured adipocytes from *PAI-1*^+/+^ or *PAI-1*^−/−^ mice, it was found that PAI-1 deletion in adipocytes promoted glucose uptake and adipocyte differentiation in response to insulin, via C/EBPα and PPARγ adipogenic transcription factors. Furthermore, PAI-1 inhibition (achieved using an anti-PAI-1 antibody) resulted in enhanced 3T3-L1 adipocyte differentiation and was associated with up-regulated expression of genes encoding C/EBPα, PPARγ and fatty acid binding protein aP2 in differentiated adipocytes. Conversely, PAI-1 overexpression in 3T3-L1 adipose cells inhibited adipocyte differentiation and was accompanied by a decrease in C/EBPα, PPARγ and aP2 production [131]. These findings indicate that PAI-1’s ability to deregulate insulin-mediated glucose uptake and adipocyte differentiation are mediated, at least in part, via the alteration of key metabolic mediators such as C/EBPα, PPARγ and aP2 in the adipose tissue (Figure 2).

To conclude, the adipokines adiponectin, leptin, resistin, IL-6, MCP-1 and PAI-1 produced by the adipose tissue are deregulated in the context of obesity. Their major roles in the metabolic, immune and vascular health are summarized in Table 1.

## 3. Adipokine Modulation by Gut Bacteria-Derived Metabolites and Suggestive Molecular Links

In the human intestine, the microbiota ecosystem, consisting of bacteria, fungi, viruses, archaea and protozoa, contributes to a large number of physiological functions such as fermentation of indigestible dietary components and vitamin synthesis, defenses against pathogens, host immune system maturation and maintenance of gut barrier functions [132]. Growing data from the literature report a major role of the gut bacterial microflora dysbiosis in the pathology of obesity and T2D. This dysbiosis is characterized by a qualitative and quantitative modification of major bacterial phyla including *Firmicutes*, *Bacteroidetes*, *Proteobacteria* and *Actinobacteria*, and results in poor bacterial diversity. This less plentiful diversity, characteristic of “obese microbiota,” was mainly associated with a lower number of bacteria of the phylum *Bacteroidetes*, especially the *Lactobacillus* genus, and an abundance of *Firmicutes* and species change in the phylum *Proteobacteria*. Host diets are extremely impactful on gut microbiota composition, altering bacteria species diversity and abundance. As a result of the bacterial ecology changes, the strong link between host and microbiota to manage host homeostasis is imbalanced, impacting host metabolism. Importantly, gut bacteria could impact obesity through (i) the intestinal permeability alteration by endotoxins that contribute to the persistent low-grade inflammation in adipose tissue, commonly observed in obesity, and (ii) the modulation of the production of bacterial metabolites that influence the gut–adipose tissue axis and alter adipokine production and action. The purposes of this chapter focus on the mechanisms by which major bacterial components such as LPS or gut bacteria-derived metabolites comprising SCFAs, TMAO and indole derivatives could modulate the adipose tissue function and adipokine production/action during obesity and gut microbiota dysbiosis.

### 3.1. LPSs and Adipokines

**Figure 3:**
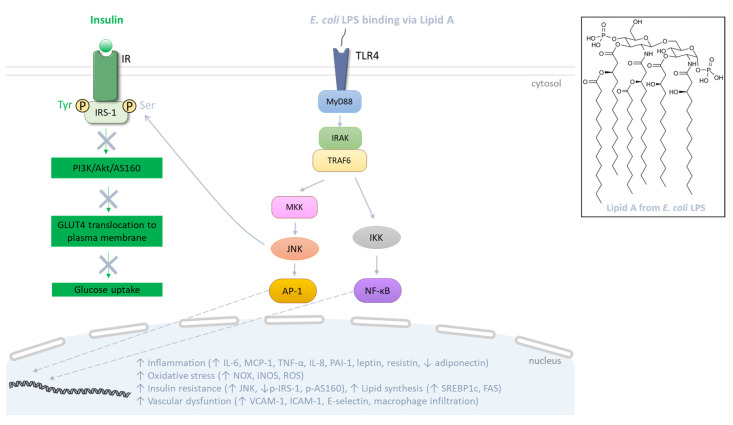
Signaling pathway mediated by *Escherichia coli* lipopolysaccharides (LPSs). At the plasma membrane of target cells, LPS binds to TLR4 that recruits MyD88, facilitating the activation of IRAK and TRAF6. Subsequently, these proteins initiate the activation of MAPK and NF-κB pathways, involving JNK and IKK, respectively. This culminates in the activation of AP-1 and NF-κB transcription factors which leads to the production of inflammatory cytokines and oxidative stress. In the adipose tissue, LPS alters the production of specific adipokines and alters lipid metabolism, thereby inciting insulin resistance. Indeed, TLR activation abrogates the insulin signaling cascade through activation of kinases like JNK that promote serine phosphorylation of IRS-1 and disruption of the PI3K/Akt/AS160 pathway responsible for GLUT4 translocation to the plasma membrane, and thus glucose uptake. In parallel, in endothelial cells, LPS promotes the production of adhesion molecules, causing macrophage infiltration.

LPSs represent the major components of the outer membrane of Gram-negative bacteria such as *Escherichia coli*, and are structurally composed of the lipid A, the core and the O antigen. Lipid A, a glucosamine-based phospholipid, constitutes the hydrophobic portion of LPS and the main pathogen associated molecular pattern (PAMP) responsible for LPS-mediated intracellular signaling (Figure 3). The core is composed of oligosaccharides linking lipid A to the O antigen, consisting of a repetition of oligosaccharides also responsible for the immunogenicity of LPS. Thus, there are different structures of LPS, depending on the nature of the phospholipids and oligosaccharides as well as the number of phosphate groups [133]. In obesity related to HFD consumption, the increase in plasma LPS level was firstly defined in 2007 by Cani et al. [15] as metabolic endotoxemia, and its contribution to adipose tissue dysfunction during obesity and related metabolic disorders is becoming more and more documented [134]. LPS translocation into the systemic circulation may be facilitated through its association with chylomicron lipoproteins involved in the intestinal lipid absorption [135,136]. Moreover, LPS may cause a pro-inflammatory environment in the gut barrier, leading to a decreased production of tight junction proteins such as claudin, occludin and zonula occludens-1 (ZO-1). This event may contribute to the loss of the gut barrier integrity, which could facilitate the paracellular passage of LPS in the bloodstream [137]. At the cellular level, LPSs mediate a pro-inflammatory signaling pathway via their binding on the plasma membrane to the innate immunity Toll-like receptors (TLRs) such as TLR4, largely studied for *E. coli* LPS (Figure 3). Indeed, the *E. coli* LPS/TLR4 interaction (via the CD14 cofactor which facilitates LPS transfer to TLR4) induces the binding of the adaptor protein myeloid differentiation primary response protein 88 (MyD88) to the receptor. In turn, MyD88 recruits the interleukin-1 receptor activated kinases (IRAK) 1 and 4, which associate with TNF receptor-associated factor 6 (TRAF6) and allow the phosphorylation of the IκB kinase (IKK) complex, and JNK, leading to the activation of the transcription factors NF-κB and AP-1, respectively [138]. Subsequently, these factors translocate into the nucleus and induce the expression of genes coding for pro-inflammatory cytokines/chemokines including IL-6, TNF-α and MCP-1. Meanwhile, the expression of genes encoding ROS-producing enzymes such as NAPDH oxidases (NOX) and iNOS is up-regulated, promoting ROS generation and oxidative stress in cells targeted by LPS (Figure 3).

Data from the literature reported higher circulating LPS levels in obese subjects (50–145 pg/mL) compared to lean subjects (50–80 pg/mL) [139,140]. Concordantly, levels of the LPS binding protein (LBP), a protein synthesized by the liver and involved in the recognition and blood transport of LPS to CD14 and TLR4, are also higher in overweight and obese individuals [141]. Of note, the plasma levels of LPS are positively correlated with intra-abdominal fat volumes, HbA1c and cardiometabolic risk factors including systolic blood pressure and fasting triglycerides, while they negatively correlate with circulating HDL cholesterol. Interestingly, a direct correlation between reduced LPS levels and improved circulating concentrations of HbA1c and triglycerides is described in obese patients after bariatric surgery [140]. Regarding the link between LPS and adipokine production from adipose tissue, Clemente-Postigo et al. [142] showed that obese subjects with high plasma LPS levels have increased mRNA levels of MCP-1 and IL-6 in visceral adipose tissue, when compared with obese subjects with lower plasma LPS levels. Circulating LPS concentration was also associated with enhanced macrophage infiltration and higher TLRs and CD14 gene expression in visceral adipose tissue. Concerning the link between metabolic endotoxemia and vascular diseases, Carnevale et al. [143] nicely demonstrated that patients with myocardial infarction have elevated levels of *E. coli* LPS in coronary thrombi, which is associated with elevated soluble P-selectin, a marker of platelet activation, and zonulin, a marker of gut permeability. Hakoupian et al. [144] recently described that subjects with ischemic stroke and intracerebral hemorrhage exhibit elevated systemic levels of LPS, LBP and CRP compared to control subjects, and the authors associated higher LPS levels with worse stroke outcomes, highlighting the potential of LPS as a risk factor for stroke.

Several preclinical studies were carried out to define the mechanistic link between the high plasma level of LPS characterizing endotoxemia and obesity-related metabolic complications. In particular, it was found that an HFD-induced obesity mouse model exhibited an elevated plasma level of LPS associated with insulin resistance development. In parallel, enhanced endotoxemia was linked to a reduced production of claudin-1 and occludin tight junction proteins, leading to gut barrier permeability [145,146]. In this model of obese mice, endotoxemia was also markedly associated with increased levels of IL-6, MCP-1 and TNF-α, decreased adiponectin levels and higher macrophage infiltration in adipose tissue [145,146,147]. Moreover, adipose tissue insulin resistance was characterized by an increased deleterious phosphorylation of IRS-1 on the serine residue, leading to insulin receptor (IR) signaling disruption. Indeed, IR signaling blockade may result from the activation by LPS of the TLR4 pathway and intermediates such as JNK are able to phosphorylate the serine residue of IRS-1, and thus can disrupt the Akt/Akt substrate (AS)160 pathway regulating GLUT4 translocation to the plasma membrane and glucose uptake [148] (Figure 3). Exposure to LPS was also shown to positively regulate lipid metabolism in adipose tissue by exacerbating the production of both SREBP1c and fatty acid synthase (FAS) proteins (Figure 3) that promote adipogenesis and fat mass gain [15,146]. Importantly, mice with inactivation of TLR4 are protected against the development of insulin resistance, and show higher levels of adiponectin and IRS-1 gene expression [149]. In rat and mouse models, LPSs also alter vascular homeostasis by increasing systemic inflammation, the production of VCAM-1 and ICAM-1 adhesion molecules and iNOS gene expression [150]. In in vitro mechanistic studies using adipocyte models, results confirm the ability of LPSs to exert pro-inflammatory activities through the involvement of the TLR4 receptor, MyD88 adaptor and NF-κB transcription factor, leading to enhanced secretion of adipokines including leptin, resistin, IL-6, MCP-1 and PAI-1 while decreasing adiponectin release [151,152]. Concomitantly, LPSs cause oxidative stress by increasing the production of ROS and pro-oxidant enzymes such as NOX2 and iNOS [151]. In vitro data also support the involvement of LPSs in the pathogenesis of insulin resistance, with evidence for the disruption of the insulin signaling pathway via the phosphorylation of JNK and a reduction in the insulin-induced phosphorylation of IRS-1 on the tyrosine residue, Akt and AS160 proteins implicated in GLUT4 translocation to the plasma membrane and glucose uptake [152,153,154]. Interestingly, the inhibition of TLR signaling pathway intermediates prevents the deleterious impact of LPS on inflammation and insulin resistance [151,153]. Similarly, in endothelial cell models, LPSs induce oxidative stress with increased ROS levels, a pro-inflammatory response through iNOS and NF-ĸB activation, as well as IL-1β, IL-6, IL-8 and IL-6 secretion [155,156], which is associated with a higher production of the adhesion molecules VCAM-1, ICAM-1 and E-selectin [155] (Figure 3). Thus, these findings provide evidence for a causal link between LPS and the up-regulated production of adipokines such as leptin, resistin, IL-6, MCP-1 and PAI-1 involved in obesity-associated inflammation, insulin resistance and vascular dysfunction, and the down-regulated production of adiponectin in favor of insulin sensitivity and vascular protection.

### 3.2. SCFAs and Adipokines

**Figure 4:**
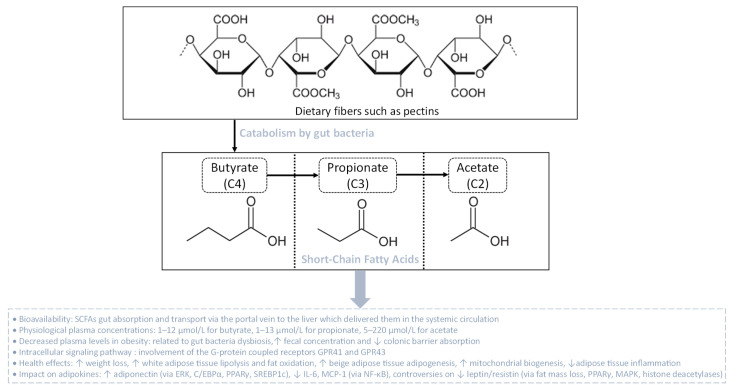
Gut bacteria catabolism of dietary fibers leading to the production of short-chain fatty acids (SCFAs). SCFAs are linear fatty acids comprising butyrate (C4), propionate (C3) and acetate (C2), resulting from the bacterial fermentation of dietary fibers. After gut absorption, SCFAs are delivered via the portal vein to reach the liver and then the systemic circulation. In the context of obesity, plasma SCFA levels decrease due to augmented fecal levels and reduced colonic barrier absorption. The physiological effects of SCFAs are mediated via G-protein coupled receptors that initiate intracellular signaling cascades, orchestrating health-enhancing impacts such as on the metabolic and endocrine functions of the adipose tissue.

SCFAs are linear fatty acids, including butyrate (C4), propionate (C3) and acetate (C2), that are metabolic by-products originating from the bacterial fermentation of dietary non-digestible complex carbohydrates (dietary fibers) such as pectins [157] (Figure 4). The production rate of these SCFAs in the colon depends on the nature and the quantity of dietary fibers consumed, and on the quantitative and qualitative presence in the colon of SCFAs-producing bacterial communities that belong to different phyla like *Actinobacteria*, *Bacteroidetes*, *Firmicutes* and *Proteobacteria* [158]. Once produced, SCFAs can be transported across the colonic barrier, reach the portal vein and the liver, and then be released in the systemic circulation [159]. Butyrate and propionate are highly extracted and metabolized by the liver, reaching an average circulating concentration of 1–12 µmol/L and 1–13 µmol/L, respectively. Oppositely, acetate is extracted at a lower rate by the liver and, thus, reaches the systemic circulation at higher concentrations (5–220 μmol/L) [160]. After entering the systemic circulation, SCFAs are able to modulate the function of different tissues such as the adipose tissue, skeletal muscle and liver, through binding to specific receptors [161]. To date, the major SCFA receptors known are the G-protein coupled receptors GPR41 and GPR43, also called free fatty acid receptor 3 (FFAR3) and FFAR2, respectively. Both GPR41 and GPR43 activate the Gi/o family proteins, although GPR43 may also signal via Gq, leading to the intracellular signaling cascades responsible for the biological effects of SCFAs (Figure 4) [162].

Clinical studies have established a positive link between fecal SCFA concentrations, obesity and obesity-associated cardiometabolic disease risks [163]. Higher fecal levels of butyrate, propionate, acetate and total SCFAs were associated with body mass index, body fat and waist circumference [164,165]. People who are overweight and obese have higher fecal SCFA concentrations than those measured in lean individuals, indicating that gut microbiota dysbiosis during obesity may lead to less efficient SCFA absorption through the colonic barrier, and therefore may promote more microbial energy harvest and SCFA fecal excretion [163]. Nonetheless, Rahat-Rozenbloom et al. [166] have reported that the change in fecal SCFA concentrations in people who are overweight and obese could not be attributed to differences in diet composition or reduced colonic SCFA absorption, but could be due to more abundant SCFA-producing bacterial communities in the colon. In parallel, Wang et al. [167] demonstrated a positive association between the plasma SCFA level and adiposity rate, supporting the idea that higher fecal excretion of SCFAs in subjects with higher body fat mass was not due to reduced colonic SCFA absorption. Thus, further studies are needed to better understand the link between the pathology of obesity and the regulation of fecal and circulating levels of SCFAs.

Besides the lack of studies comparing the circulating and peripheral tissue concentrations of SCFAs during obesity, it is known that SCFAs affect the metabolism of the adipose tissue, skeletal muscle and liver. Indeed, animal studies show that SCFA administration could reverse or reduce body weight gain and adiposity growth [168,169]. In an HFD-induced obesity mouse model, sodium butyrate supplementation at 5% wt/wt leads to weight loss, by boosting energy expenditure and fat oxidation [169]. Likewise, supplementation of SCFAs causes significant changes in the *Firmicutes*/*Bacteroidetes* ratio and elevates the expression of genes encoding GPR43 and GPR41 in adipose tissue while reducing the levels of both receptors that are found in the colon [169]. The effects of SCFAs on the GPR expression rate and gut microbiota composition were associated with body weight reduction and enhanced lipolysis and free fatty acid oxidation in adipose tissue, promoting beige adipogenesis, mitochondrial biogenesis and inhibiting chronic inflammation. However, HFD-fed mice lacking both GPR41 and GPR43 were found to exhibit improved glucose tolerance and insulin sensitivity, showing that the role of GPR41 and GPR43 during obesity still needs to be clarified [170]. In vitro cellular studies demonstrate that SCFAs differentially affect lipolysis, in a SCFA type-dependent and dose-dependent manner. Acetate (1–1000 µM), propionate (10 mM) and butyrate (10 mM) lead to an antilipolytic profile through GPR, by attenuating the phosphorylation of hormone-sensitive lipase (HSL) at phosphor-Ser650 and phosphor-Ser562 in differentiated human multipotent adipose-derived stem cells, and in rat primary adipocytes, respectively [171,172]. Likewise, a dose of 4 mM acetate decreases the phosphorylation of HSL_(Ser563)_ and the rate of lipolysis in murine 3T3-L1 adipocytes [173]. Interestingly, acetate and propionate at physiological concentrations (100–300 µM) suppress lipolytic activity by up to 50% via GPR43 [174]. In contrast, Rumberger et al. [175] showed that in a long-term experiment using 20 mM propionate and 5 mM butyrate, SCFAs induce increased lipolysis rates in 3T3-L1 adipocytes. The relationship between SCFAs and adipocyte lipid metabolism still needs to be elucidated [176], with further studies in humans to better understand the mechanistic pathways by which SCFAs affect lipolysis and subsequent effects on the adipose tissue metabolism. The next part of this review focuses on data from the literature describing the links between SCFAs and key adipokines during obesity.

#### 3.2.1. SCFAs, Adiponectin and Resistin

Data from the literature describing the effect of SCFAs on the inflammatory profile of adipose tissue are a bit controversial, with a few studies showing pro-inflammatory effects and others demonstrating anti-inflammatory actions. However, it is mostly reported that SCFAs exert anti-inflammatory properties. Indeed, based on animal models, it is currently known that adiponectin levels are low in HFD-induced obese mice, contributing to adipose tissue pro-inflammatory status. Interestingly, butyrate restores the production of adiponectin and is able to protect the adipose tissue from leukocyte infiltration, alleviating inflammation [177]. Thus, SCFAs may counteract the infiltration of immune cells and overall inflammation-associated complications in obese adipose tissue [178]. Interestingly, HFD-induced obese mice with SCFA supplementation have increased adiponectin and resistin mRNA levels. It was found that SCFAs could modulate adiponectin and resistin gene expression through DNA methylation and may correct aberrant expressions of adiponectin and resistin by epigenetic regulation that might be helpful for obesity prevention. Similar results have been shown by modulating gut microbiota with antibiotics which counteract gut bacteria dysbiosis, thereby promoting reduced SCFA fecal contents. Nevertheless, the regulation of resistin gene expression in obesity needs further investigation because of controversial studies [179,180]. An in vitro study on 3T3-L1 preadipocytes demonstrated that mRNA levels of GPR43 and PPARγ were increased by acetate at 1 mM [181]. Concomitantly, it was found that butyrate administration stimulates adipocyte differentiation and adiponectin expression in porcine stromal vascular cells by inducing the expression of genes coding for C/EBPα, PPARγ and SREBP-1c. These results indicate that SCFAs are strong regulators of adipogenesis and adiponectin gene expression, probably via the interaction between SCFAs and GPR41/43. Accordingly, it was demonstrated that SCFA and GPR43 binding inhibits cAMP production and increases intracellular Ca^2+^ levels, which leads to activation of the MAPK pathway, particularly via the extracellular signal-regulated kinase (ERK) arm of the pathway [182]. ERK activation has been shown to be required for adipogenesis in cultured adipocytes and enhances the activity of factors that regulate both C/EBPα and PPARγ expression [183].

#### 3.2.2. SCFAs and Leptin

SCFAs have been shown to regulate the production of leptin. Indeed, based on animal models, two studies demonstrated that resistant starch and butyrate supplementation led to inverse results for leptin levels and GPR activation. As expected, GPR43 and GPR41 expression increased in adipose tissue in response to increased SCFA. However, serum leptin concentrations decreased in animals supplemented with butyrate and resistant starch compared to HFD-fed animals, which contrasts with results obtained in vitro [184,185]. Jia et al. [185] demonstrated that oral administration of sodium butyrate at 80 mg every day for 10 days increased GPR43 expression, even though the supplementation caused both weight and adiposity loss. Therefore, these findings assume that leptin expression is more dependent on fat mass than on the direct effect of GPR-activating SCFAs in adipocytes. SCFAs can influence the expression of GPR in adipose tissue, but leptin production in animal models appears to be mainly related to adiposity rather than to the presence of SCFAs in adipose tissue. SCFA production through gut bacteria modulation or SCFA supplementation seems to be related to reduced leptin levels through pathways, such as AMPK, PPAR-γ and histone deacetylases, that suppress weight gain via activation of lipolysis and/or promotion of adipocyte differentiation [186,187,188]. However, food intake was not measured in most of these studies and did not explain if adiposity and leptin levels are modulated by either food intake or SCFAs [181,185,189]. Oppositely, it was reported that 3 mM of butyrate increased the level of leptin in primary cultures of mouse white adipose tissue and that oral administration of 500 μmoles of propionate in bolus by gavage elevated plasma leptin levels [176]. Furthermore, it was demonstrated that dietary supplementation with a mixture of SCFAs or acetate increases leptin expression while reducing MCP-1 and IL-6 mRNA in the white adipose tissue of HFD-fed mice, arguing the fact that the results from in vivo studies are controversial and need further investigations [176].

Despite controversial results from in vivo studies regarding leptin regulation by SCFAs, most of the in vitro studies demonstrated that SCFAs stimulate leptin expression through activation of GPR41, which is highly expressed in adipose tissue. It was found that 3 mM propionate increases leptin release through the activation of GPR41 in primary adipocyte cultures [176]. Simultaneously, 3 mM propionate mitigates the expression of resistin via GPR43 [190]. GPR43 is less expressed in adipocytes, thus it remains unclear if GPR43 plays a role in leptin expression. Nevertheless, a study conducted by Zaibi et al. [191] revealed that while acetate has an inhibitory effect upon leptin production, 3 mM propionate and 0.2 mM butyrate positively enhance the release of this adipokine in murine adipocytes, mediated by GPR43 activation. Taken together, these findings raise the possibility that either GPR41 or GPR43 modulates SCFA-stimulated leptin production in white adipose tissue. However, the different effects observed in adipose tissue cellular models can be discussed depending on the SCFAs that may act differently based on the activated receptor, and the dose used, which is extra-physiological and rarely achievable by endogenous fermentation.

#### 3.2.3. SCFAs and Other Adipokines

The potential of butyrate to mitigate MCP-1, IL-6 and TNF-α secretion in co-cultured 3T3-L1 adipocytes and RAW264.7 macrophages, in a dose-dependent manner, was reported. Butyrate inhibited the phosphorylation of MAPKs, the activity of NF-κB in co-cultured macrophages and suppressed lipase activity in co-cultured adipocytes. Lipase inhibitors significantly attenuated the production of TNF-α, MCP-1 and IL-6 in the co-culture medium as effectively as butyrate. Butyrate suppressed the protein production of adipose triglyceride lipase, HSL and fatty acid-binding protein 4 in co-cultured adipocytes. Pertussis toxin, which is known to block GPR41 completely, inhibited the anti-lipolysis effect of butyrate. Conversely, the SCFAs propionate and butyrate improved adipose tissue inflammation. Propionate may have a direct beneficial effect on adipose tissue in overweight subjects, as it reduced the mRNA level and secretion of inflammatory cytokines [192]. Recently, it has been shown that a reduction in the levels of gut butyrate generates local inflammation and foam cell formation, contributing to gut barrier disruption and favoring bacterial translocation including mobilization of LPS and TMAO [181].

### 3.3. TMAO and Adipokines

**Figure 5:**
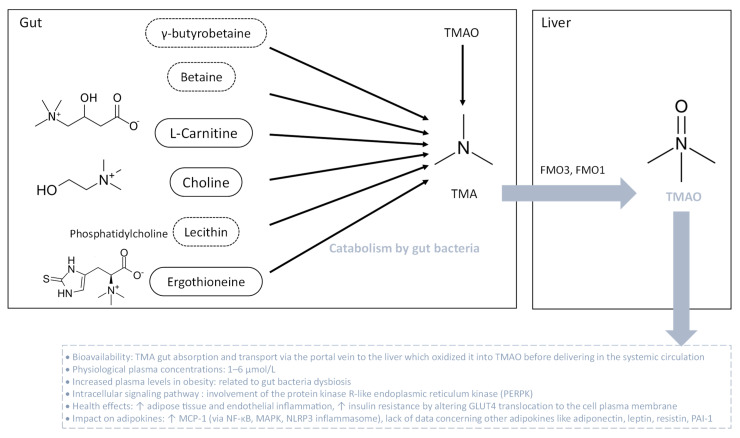
Bacterial degradation of trimethylamine (TMA) for trimethylamine N-oxide (TMAO) formation. TMA is generated when the gut microbiota metabolize dietary carnitine, choline and choline-containing compounds. After gut absorption, at the hepatic level, TMA undergoes conversion into TMAO through flavin-dependent monooxygenase (FMO) isoforms 1 and 3. Then, TMAO enters the systemic circulation. During obesity, plasma TMAO levels are elevated. TMAO is associated with adipose tissue and endothelial inflammation, along with insulin resistance. The intracellular signaling pathway initiated by TMAO involves protein kinase R-like endoplasmic reticulum kinase (PERPK), leading to a pro-inflammatory status.

TMAO is produced by the liver from TMA which is generated by the bacterial fermentation in the gut principally from diets containing betaine, L-carnitine, choline, phosphatidylcholine (lecithin) and other choline-containing compounds mostly found in eggs and meats (Figure 5). TMA can be found also in some foods, in a natural way. In the gut, the main TMA-producing bacteria are *Clostridium* spp., *Eubacterium* spp. and *Gammaproteobacteria*, primarily *E. coli* [193]. Nevertheless, TMA-producing bacteria in the human intestine are still to be determined regarding the lack of knowledge in this field. TMA, ingested or formed in the gut, is rapidly absorbed by passive diffusion into the portal circulation and then oxidized in the liver to TMAO by the action of flavin-containing monooxygenases FMO3 and FMO1. The main enzyme responsible for the conversion of TMA into TMAO in the liver is FMO3, which has ten-fold higher specific activity than does FMO1. Both humans and mice exhibit a sexual dimorphism of hepatic FMO3 expression which is reduced in males [194]. Once oxidized in the liver, TMAO is transported into the systemic circulation via passive diffusion in the concentration range of 1–6 µM [195,196,197]. TMAO is strongly associated with pro-inflammatory events, vascular inflammation, atherosclerosis, vascular calcification and cardiovascular disease (Figure 5) [195,197,198,199,200,201,202]. 

Clinical evidence revealed that circulating TMAO levels are increased along with BMI during obesity, ranging from approximately 8 µM for people who are overweight to more than 12 µM for people with a BMI of 40 or higher. Furthermore, a positive correlation between circulating levels of TMAO and obesity markers, blood pressure, metabolic profile and T2D was established [203,204]. Croyal et al. [205] correlated plasma TMAO concentrations and diabetes-related covariates such as renal function, HDL-cholesterol and soluble TNF-α receptor concentrations, which characterize systemic inflammation. Evidence from animal studies showed that plasma TMAO level is positively correlated to fat mass gain after 8 weeks of HFD in mice. Of note, altered production of FMO3 by antisense oligonucleotide protects mice from HFD-induced obesity, supporting the strong link between TMAO and obesity [204]. Concerning the impact of TMAO on adipose tissue, a phosphoproteomic analysis demonstrated that TMAO alters the phosphorylation of proteins such as synaptosome-associated 23 kDa protein (SNAP 23), involved in GLUT4 vesicle binding to the cell membrane for insulin response [206]. In HFD-induced obese mice, TMAO promotes adipose tissue inflammation by increasing the production of MCP-1 and decreasing the synthesis of anti-inflammatory cytokines. Such events coincide with insulin resistance and chronic low-grade inflammation [207]. However, there is still a lack of data regarding the effect of TMAO on adiponectin, leptin and resistin production, and molecular links between TMAO and inflammation in adipocytes. In parallel, the impact of TMAO on vascular inflammation is much more well-known. Indeed, TMAO induces vascular inflammation by activating NF-κB and MAPK signaling pathways [198,200] as well as the inflammasome sensor protein NLRP3 from the nucleotide-binding oligomerization domain, leucine-rich repeat-containing proteins family [199] in endothelial cells. Data from the literature have established a crosstalk between those signaling pathways contributing to pro-inflammatory cytokine production [208]. Interestingly, one robust study determined that TMAO directly binds to the protein kinase R-like endoplasmic reticulum (ER) kinase (PERK), an ER stress response protein [209]. It was demonstrated that both TMAO and PERK activate the NF-κB, MAPK and NLRP3 inflammasome [210]. Thus, TMAO could promote pro-inflammatory adipokine production by activating the NF-κB, MAPK and NLRP3 inflammasome, via its direct binding to PERK. Although some mechanistic links are cited, further studies are needed to determine TMAO’s contribution to adipocyte dysfunction and adipokine production during obesity.

### 3.4. Indole Derivatives and Adipokines

**Figure 6:**
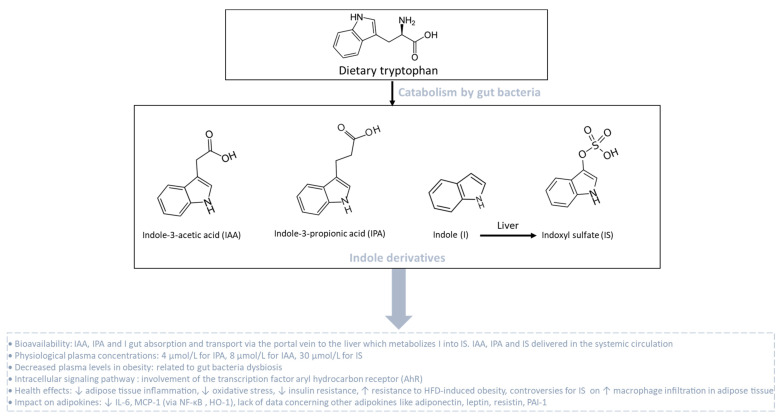
Catabolism of dietary tryptophan by gut bacteria leading to the production of indole derivatives. Dietary tryptophan undergoes catabolism by gut bacteria, giving rise to the synthesis of indole derivatives such as indole-3-acetic acid (IAA), indole-3-propionic acid (IPA) and indole (I). Indole derivatives are transported via the portal vein to the liver. In the liver, indole (I) is metabolized into indoxyl sulfate (IS). During obesity associated with gut bacteria dysbiosis, plasma levels of indole derivatives are reduced. Tryptophan-derived catabolites act as agonists for the aryl hydrocarbon receptor (AhR) that improves the metabolic and endocrine function of adipose tissue.

Indole derivatives are produced from the gut bacterial metabolism of tryptophan, which is one essential amino acid provided by the diet. Tryptophan metabolism appears now as a key modulator of the gut microbiota impacting major physiological and pathological pathways [211]. In homeostatic conditions, tryptophan metabolism in the intestine follows different pathways including one from the direct transformation of tryptophan by the gut microbiota, representing 4–6% of tryptophan metabolism. Tryptophan can be decarboxylated into tryptamine to produce indole acetic acid (IAA). Tryptophan can also be transformed by a tryptophanase into indole-3-propionic acid (IPA), indole and indoxyl sulfate (IS), which is produced in the liver from indole (Figure 6). Still, other indole derivatives exist but will not be presented in this part of the review. Phyla that produce tryptophan-derived metabolites include *Actinobacteria*, *Bacteroidetes*, *Firmicutes* and *Proteobacteria*. In homeostatic conditions, circulating levels of these metabolites reach 4, 8 and 30 µM for IPA, IAA and IS, respectively. These groups of metabolites may affect host physiology through the transcription factor aryl hydrocarbon receptor (AhR) by modulating multiple cellular pathways [212] involved in the regulation of inflammation, oxidative stress, the insulin response and fat storage (Figure 6).

Cussotto et al. [213] compared circulating levels of tryptophan-derived metabolites in people with or without obesity and demonstrated an altered tryptophan metabolism with a significant reduction in IPA (1.5 µM), IAA (5.5 µM) and IS (12 µM) in patients with obesity. Of note, levels of high-sensitivity (hs) CRP and hsIL-6, used as markers of systemic inflammation, were correlated positively with alterations in tryptophan metabolic pathways. These results suggest that changes in bacterial tryptophan metabolic pathways, due to a low flora richness in patients with obesity, could drive obesity-related systemic inflammation. Meanwhile, higher circulating levels of indole derivatives such as IPA have been strongly correlated with high microbiome diversity, dietary fiber intake and a lower risk of several metabolic syndrome parameters such as a better insulin secretion and T2D. Furthermore, IPA levels were negatively correlated with low-grade inflammation, and the reduction in T2D risk might be mediated by the interplay between dietary fiber intake and inflammation or by direct effect of IPA on preserving β-cell function [214,215,216]. Using clinical and pre-clinical studies, Natividad et al. [217] demonstrated that metabolic syndrome is associated with the reduced capacity of the microbiota to metabolize tryptophan into derivatives that are able to activate AhR. Nevertheless, previous studies reported contradictory results, suggesting that AhR can be deleterious and plays a large and broad role in obesity and associated complications [218,219]. Moreover, AhR^−/−^ mice fed with HFD were partially protected against diet-induced glucose intolerance, suggesting a potential role of AhR in insulin resistance [219]. Interestingly, a recent study evaluating the role of AhR specifically in adipocytes showed that adipocyte-specific AhR KO mice are resistant to HFD-induced obesity and partially protected against insulin resistance [220]. There is still a lack of data concerning the impact of indole derivatives on the modulation of adiponectin, leptin, resistin and PAI-1 production in adipose tissue during obesity. Using RAW264.7 macrophages, Ji et al. [221] reported the protective effect and underlying mechanism of IAA against LPS-induced inflammatory response and free radical generation. Indeed, IAA at 1000 µM significantly reduced LPS-induced expression of IL-6 and MCP-1 as well as ROS and nitric oxide (NO) generation, by activating heme oxygenase-1 (HO-1), an enzyme with antioxidant and anti-inflammatory properties. Interestingly, this effect was dependent on NF-ĸB mitigation but not AhR, suggesting that other mechanistic events are involved. Conversely, IS seems to be much more deleterious. In 3T3-L1 mature adipocytes, IS at 1 mM induced MCP-1 gene expression and ROS production through the organic anion transporter/NADPH oxidase/ROS pathway. IS increased macrophage infiltration, verified by co-culturing 3T3-L1 adipocytes and mouse macrophage cells, resulting in an increase in adipose tissue inflammation [222]. Nonetheless, the doses used in those cellular models are supra-physiological and rarely achievable by gut bacterial metabolism. Thus, there is still a debate concerning the role of tryptophan-derived metabolites and AhR on adipokine modulation in adipose tissue during obesity.

## 4. Conclusions

**Figure 7:**
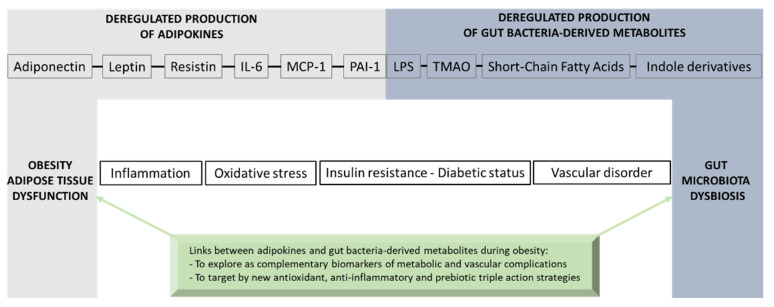
Overview of the molecular bridge based on adipokines and gut bacteria-derived metabolites that links adipose tissue dysfunction and obesity to gut microbiota dysbiosis.

Adipose tissue exerts multifunctional activities related to lipid storage, thermal activity and mechanical insulation, as well as endocrine functions that are essential for whole-body energy homeostasis via the production of key adipokines such as adiponectin, leptin, resistin, IL-6, MCP-1 and PAI-1. During obesity, adipokine production imbalance occurs and promotes chronic-low grade inflammation, oxidative stress, insulin resistance, T2D onset and vascular complications. A causal link between obesity and gut microbiota dysbiosis has been demonstrated. The deregulation of gut bacteria communities characterizing this dysbiosis leads to an altered production of gut bacteria-derived substances including LPS and specific metabolites such as SCFAs, TMAO and indole derivatives that have a strong interplay in the modulation of adipokines. If some of these bacterial metabolites may exert beneficial health effects, such as SCFAs and indole derivatives, others like LPS and TMAO may play detrimental roles leading to inflammation, oxidative stress and insulin resistance in the context of obesity and vascular complications. Nevertheless, there are still some controversies concerning the effects of bacterial metabolites on adipose tissue function and adipokine modulation due to the high doses used in the experimental models. Moreover, the mechanisms by which bacterial metabolites modulate adipokine production, as well as the metabolic and vascular health, need further well-controlled studies. Figure 7 gives an overview of the molecular bridge based on adipokines and bacterial metabolites, which links obesity to gut bacteria dysbiosis. Given the physiological importance of the gut–adipose tissue axis, a better understanding of the molecular links between adipokines and gut bacteria-derived metabolites could bring new complementary clinical biomarkers in the context of obesity. The validation of innovative dietary/pharmacological strategies which are able to exert a triple action via antioxidant effects, anti-inflammatory properties and modulatory roles of the gut microbiota dysbiosis, such as prebiotics, may help to reduce the burden of obesity and its metabolic and vascular complications.

## Figures and Tables

**Table 1:** Roles of adipokines in the metabolic, immune and vascular health.

Adipokines	Roles and Abilities to Induce (↑) or Attenuate (↓) Molecular Events	References
Adiponectin	Physiological plasma levels range from 3 to 30 μg/mL in humans Low levels are associated with metabolic and vascular disorders	[30,31,32,34,35,36]
	Anti-inflammatory and antioxidant effects (↓ NF-κB, MCP-1, IL-6, IL-8, TNF-α, iNOS)	[39,40]
	Pro-adipogenic action (↑ C/EBPα, PPARγ, SREBP1c) Insulin-sensitizing effect (↑ GLUT4 and glucose uptake)	[39,40]
	Vasoprotective role (↑ NO, eNOS phosphorylation, ↓VCAM-1, ICAM-1, E-selectin)	[41,42]
Leptin	Higher plasma levels in patients with obesity (~36 ng/mL) than in healthy subjects (8 ng/mL) High levels during obesity increase cardiovascular disease risk	[48,49,50,52,53]
	Low levels improve adipose tissue inflammation, insulin sensitivity and glucose tolerance	[55]
	Pro-inflammatory effect (↑ p38 MAPK, JAK/STAT, JNK pathways)	[56,57,58]
	Induction of oxidative stress (↑ ROS production)	[58]
Resistin	Higher plasma levels in patients with obesity (~15 ng/mL) than in lean subjects (<10 ng/mL) High resistinemia correlates with an increased risk of all-cause and cardiovascular death	[59,65,66,67]
	Causal role in insulin resistance (↓ activation of IRS-1, ↓ glucose uptake)	[68,72]
	Induction of inflammatory and vascular disorders (↑ ET-1, MCP-1, ICAM-1, VCAM-1)	[73,74]
IL-6	Plasma levels increase from ~2.5 pg/mL in lean subjects to 4.4 pg/mL in patients with obesity	[81]
	Pro-inflammatory effect (↑ macrophage infiltration in white adipose tissue)	[89]
	IL-6-deficient mice develop late-onset obesity and systemic glucose intolerance	[91]
	Reduction in insulin sensitivity and insulin-dependent hepatic glycogen synthesis	[85]
MCP-1	Higher plasma levels in patients with obesity (220 pg/mL) than in lean subjects (100 pg/mL)	[102,103]
	Pro-inflammatory effect (↑ macrophage infiltration)	[109,110]
	Elevation of insulin resistance (↓ glucose uptake, ↓ GLUT4, PPAR-γ)	[111]
	Induction of vascular disorders (HDL dysfunction, cholesterol efflux alteration)	[112]
PAI-1	Higher plasma levels in patients with obesity (55.1 ng/mL) than in lean subjects (10.4 ng/mL)	[120,122]
	Pro-inflammatory effect (↑ macrophage infiltration in the white adipose tissue)	[126,127]
	Anti-adipogenic action (↓ C/EBPα, PPARγ, aP2), insulin resistance induction	[131]
	Causal role in the development of atherosclerosis	[128,129]

## Data Availability

Not applicable.
